# Angiotensin II-Induced Cardiac Effects Are Modulated by Endocannabinoid-Mediated CB_1_ Receptor Activation

**DOI:** 10.3390/cells10040724

**Published:** 2021-03-24

**Authors:** Zsuzsanna Miklós, Dina Wafa, György L. Nádasy, Zsuzsanna E. Tóth, Balázs Besztercei, Gabriella Dörnyei, Zsófia Laska, Zoltán Benyó, Tamás Ivanics, László Hunyady, Mária Szekeres

**Affiliations:** 1 Institute of Translational Medicine, Semmelweis University, 1094 Budapest, Hungary; dina.wafa.93@gmail.com (D.W.); besztercei.balazs@med.semmelweis-univ.hu (B.B.); zsofi.laska@gmail.com (Z.L.); benyo.zoltan@med.semmelweis-univ.hu (Z.B.); ivanics.tamas@med.semmelweis-univ.hu (T.I.); 2 Department of Physiology, Semmelweis University, 1094 Budapest, Hungary; nadasy.gyorgy@med.semmelweis-univ.hu (G.L.N.); hunyady.laszlo@med.semmelweis-univ.hu (L.H.); 3 Department of Anatomy, Histology and Embryology, Semmelweis University, 1094 Budapest, Hungary; toth.zsuzsanna.emese@med.semmelweis-univ.hu; 4 Department of Morphology and Physiology, Semmelweis University, 1088 Budapest, Hungary; dornyeig@se-etk.hu; 5 Laboratory of Molecular Physiology, Semmelweis University and Hungarian Academy of Sciences, 1094 Budapest, Hungary

**Keywords:** Angiotensin II, cardiac, endocannabinoid, CB_1_ cannabinoid receptor, myocardial function, coronary flow, vasoconstriction

## Abstract

Angiotensin II (Ang II) has various cardiac effects and causes vasoconstriction. Ang II activates the type-1 angiotensin receptor—G_q/11_ signaling pathway resulting in the release of 2-arachidonoylglycerol (2-AG). We aimed to investigate whether cardiac Ang II effects are modulated by 2-AG-release and to identify the role of type-1 cannabinoid receptors (CB_1_R) in these effects. Expression of CB_1_R in rat cardiac tissue was confirmed by immunohistochemistry. To characterize short-term Ang II effects, increasing concentrations of Ang II (10^−9^–10^−7^ M); whereas to assess tachyphylaxis, repeated infusions of Ang II (10^−7^ M) were administered to isolated Langendorff-perfused rat hearts. Ang II infusions caused a decrease in coronary flow and ventricular inotropy, which was more pronounced during the first administration. CB agonist 2-AG and WIN55,212-2 administration to the perfusate enhanced coronary flow. The flow-reducing effect of Ang II was moderated in the presence of CB_1_R blocker O2050 and diacylglycerol-lipase inhibitor Orlistat. Our findings indicate that Ang II-induced cardiac effects are modulated by simultaneous CB_1_R-activation, most likely due to 2-AG-release during Ang II signalling. In this combined effect, the response to 2-AG via cardiac CB_1_R may counteract the positive inotropic effect of Ang II, which may decrease metabolic demand and augment Ang II-induced coronary vasoconstriction.

## 1. Introduction

The renin-angiotensin-aldosterone system (RAAS) plays a key regulatory role in cardiovascular and salt-water homeostasis. Angiotensin II (Ang II) is the main effector molecule of the RAAS. Ang II is an octapeptide hormone produced by the angiotensin-converting enzyme. It plays a crucial role in numerous physiological and pathological processes involving aldosterone secretion, cell-proliferation, inflammation and atherosclerosis [1,2,3]. In the cardiovascular system, its peripheral vasoconstrictor and central pressor effects lead to the elevation of systemic blood pressure [3]. Direct short-term cardiac effects of Ang II include a decrease in coronary flow (CF) and controversial effects on contractility [4,5,6,7,8,9]. Moreover, Ang II produced by the locally activated RAAS has adverse effects on the remodelling process of the vascular and cardiac tissues in various pathophysiological conditions [3,10].

Ang II activates type-1 and type-2 angiotensin receptors (AT_1_R and AT_2_R), among which AT_1_R mediates its major short-term and long-term actions [2]. Ang II binding to AT_1_R causes AT_1_R interaction with heterotrimeric G proteins, including G_q/11_, G_12/13_, and G_i_ [2,11]_._ Coupling to G_q/11_ classically leads to phospholipase C stimulation and activation of downstream inositol-triphosphate and diacylglycerol dependent pathways [2]. Additionally, Ang II-mediated G_q/11_ activation-dependent signalling can lead to endocannabinoid formation and release [12,13].

Endocannabinoids (i.e., endogenous cannabinoids, such as arachidonoyl ethanolamide (anandamide) and 2-arachidonoylglycerol (2-AG) are produced by several cell types, and by acting on type-1 and type-2 cannabinoid receptors (CB_1_R and CB_2_R), they exert diverse biological effects [14,15,16,17,18,19] including tissue-specific local regulatory mechanisms [20]. In the cardiovascular system, negative inotropic, hypotensive and cardioprotective actions of cannabinoids have been previously reported [21,22,23,24,25,26,27], in which endothelium-derived nitric oxide and effects from perivascular nerves are involved [18,28].

We have previously reported that activation of AT_1_R can cause 2-AG-mediated paracrine transactivation of CB_1_R in cells expressing both AT_1_ and CB_1_ receptors. Ang II-induced CB_1_R activation was inhibited by diacylglycerol-lipase (DAGL) inhibitors, suggesting that diacylglycerol is converted to 2-AG by DAGL during the signalling of calcium-mobilizing hormones [29]. We also investigated this mechanism in the vascular tissue and found that the vasoconstrictor effect of Ang II was attenuated via G_q/11_-mediated vascular endocannabinoid formation [15]. 

Cardiac AT_1_R activation has utmost relevance in heart (patho)physiology [3,30,31,32]; however, it is unknown whether concomitant 2-AG production and CB_1_R activation modulate these effects [29]. The main aim of this study was to reveal the participation of paracrine endocannabinoid mechanisms in Ang II signalling in the heart. For this purpose, we investigated how inhibition of 2-AG formation and CB_1_R activation modify the effects of Ang II administration in isolated Langendorff-perfused rat hearts. As short-term actions of both Ang II and 2-AG in the heart are complex and controversial [4,5,6,7,8,9], we first characterized the responses of the isolated rat heart to these mediators in our experimental setting. 

## 2. Materials and Methods

### 2.1. Animals 

The experiments were performed on isolated hearts of adult male Sprague-Dawley rats weighing 300–350 g. Animals were bred and housed in the animal facility at Semmelweis University, kept in a 12/12-h diurnal cycle with free access to water and standard rat chow. All applied procedures conform to the guidelines of the Hungarian Law of Animal Protection (28/1998) and were approved by the Government Office of Pest County (Permission number: PEI/001/820-2/2015 and PE/EA/1428-7/2018).

### 2.2. Langendorff Heart Preparation 

Animals were anesthetised by intraperitoneal injection of 40 mg/kg pentobarbital (Euthasol 40%; Produlab Pharma BV, Raamsdonksveer, The Netherlands) before the excision of the heart. The isolated hearts were cannulated and perfused using a gravitational Langendorff apparatus (Experimetria Ltd., Budapest, Hungary) at constant, 70 mmHg pressure with a modified Krebs–Henseleit buffer. A detailed description of the perfusion and methodology of measuring hemodynamic parameters in a Langendorff-perfused heart preparation was given previously [33,34,35]. Briefly, left ventricular pressure (LVP) was recorded using a fluid-filled balloon catheter introduced into the ventricle and connected to a pressure gauge (Experimetria Ltd., Budapest, Hungary), whereas coronary flow (CF) was continuously monitored with a transit-time flow probe placed into the inflow line (Transonic 2-PXN flow probe, TS410-tubing flow module Transonic Systems Inc., Ithaca, NY, USA) [33,34,35].

Data acquisition and analysis were performed using the Haemosys software (Experimetria Ltd., Budapest, Hungary). Left ventricular developed pressure (LVDevP) was calculated as the difference between peak systolic and minimum diastolic pressures. The positive and negative maximum values of the first derivative of the LVP (+dLVP/dt_max_, −dLVP/dt_max_) were determined as indices of left ventricular contractile and lusitropic performance, respectively [33,34,35].

### 2.3. Experimental Protocol

After cannulation of the isolated heart, a 30-min equilibration period was allowed. Afterward, baseline data were recorded, and the respective substances or their vehicles were infused as described previously [33]. 

To describe the effects of Ang II on the CF and contractile function of isolated Langendorff-perfused rat hearts, we obtained concentration-response relationship curves by infusing Ang II (Sigma-Aldrich, Budapest, Hungary) dissolved in calcium-free Krebs–Henseleit buffer in increasing concentrations (10^−9^, 3 × 10^−9^, 10^−8^, 10^−7^ M). Each concentration was administered for 3 min. 

We also assessed the effects of repeated Ang II treatments. For this purpose, we infused Ang II repetitively four times, each time for 3 min at a concentration of 10^−7^ M. Between the consecutive infusion periods, the hearts were allowed to equilibrate for 10 min. During this period, all measured parameters returned to control values. 

The endocannabinoid 2-AG (Sigma-Aldrich, Budapest, Hungary) and the CB receptor agonist WIN55,212-2 (Sigma-Aldrich, Budapest, Hungary) were infused into isolated hearts for 5 min at a concentration of 10^−6^ M to assess the influence of CB receptor activation on CF and heart function. 2-AG administration was also repeated in the presence of the CB_1_R inhibitor O2050 (10^−6^ M Cayman Chemicals, Ann Arbor, MI, USA). After the first 2-AG infusion, the heart was allowed to equilibrate for 10 min. O2050 infusion was initiated 5 min before the second 2-AG infusion, then continuously coadministered during agonist treatment. 

In order to assess the role of the CB_1_R and DAGL activation in mediating the effects of Ang II, Ang II was administered in the presence of the CB_1_R inhibitor O2050 (10^−6^ M Cayman Chemicals, Ann Arbor, MI, USA) and the DAGL inhibitor Orlistat (10^−5^ M) (tetrahydrolipstatin, Sigma-Aldrich, Budapest, Hungary) or their vehicles in separate experiments. In these protocols, after the first Ang II (10^−7^ M) infusion, Ang II (10^−7^ M) was repeatedly applied together with the antagonists. Each Ang II infusion lasted for 3 min, and the cardiac parameters were allowed to return to control values between the first and second infusion protocols. 

### 2.4. Immunohistochemistry

Isolated hearts of 2 rats and 2 mice (CB_1_R knockout (−/−, CB_1_R-KO) and wild type (+/+, C57BL/6, Cnr1tm1zim) mice (25–30 g) which were kindly provided by Professor Andreas Zimmer, University of Bonn [36]), were fixed in 4% paraformaldehyde right after excision for 24 h and then placed in 10 and 20% sucrose solutions (15–18 h each) for cryoprotection. The tissues were quickly frozen on dry ice as described previously [37].

Cryostat sections (12-µm-thick cross-sections from the heart tissue) were mounted on Super Colorfrost slides (Thermo Fisher Scientific, Waltham, MA, USA). Sections were blocked with 1% bovine serum albumin (BSA) for 15 min. Endogenous peroxidase activity was eliminated using 3% H_2_O_2_ solution for 15 min; immunostaining was performed by applying CB_1_R primary antibody (1:500, Cayman Chemicals, Ann Arbor, MI, overnight). The reaction was developed using the standard ABC method (Vector Labs, Burlington, CA, USA). Diaminobenzidine was used for visualization.

### 2.5. Immunofluorescent Visualization of CB1 Receptors in Rat Cardiac Tissue

Two rat hearts fixed in 10% neutral-buffered formalin were dehydrated, embedded in paraffin, ~2 µm thick serial sections were cut, mounted on silanized glass slides, and kept in a thermostat at 65 °C for 1 h. Sections were dewaxed and rehydrated. For antigen retrieval, heating for 20 min in Tris-ethylenediaminetetraacetic acid (EDTA) buffer pH 9.0 (0.1 M Tris-base and 0.01 M EDTA) using an Avair electric pressure cooker (ELLA 6 LUX(D6K2A), Bitalon Ltd., Pécs, Hungary) followed by a 20 min cooling with an open lid was applied. Non-specific proteins were blocked in 3 % bovine serum albumin (BSA, #82-100-6, Millipore, Kankakee, IL, USA) diluted in 0.1 M Tris-buffered saline (TBS, pH 7.4) containing 0.01% sodium-azide for 30 min [38]. The sections were incubated with the primary antibodies (Anti-Troponin T (Cardiac Muscle) clone 9C2.1 (mouse), 1:50 dilution, Sigma-Aldrich; and CB1 Receptor Polyclonal Antibody (rabbit), 1:250 dilution, Cayman Chemicals, Ann Arbor, MI, USA) diluted in 1% BSA/TBS+TWEEN (TBST, pH 7.4) for 2 h. Afterward, Alexa 546 goat anti-mouse (Thermo Fisher Scientific, Waltham, MA, USA) and Alexa 488 goat anti-rabbit (Thermo Fisher Scientific, Waltham, MA, USA) secondary fluorescent antibodies (dilution 1:100) were used for 1h incubation. Nuclei were counterstained with 4′,6-diamidino-2-phenylindole (DAPI) (Thermo Fischer Scientific, Waltham, MA, USA; 1:100) for 10 min. Stained sections were covered with aqueous mounting medium (BrightMountPlus, ab103748, Abcam). Slides were imaged using an inverted microscope (Nikon Ti2) equipped with a 60× oil immersion objective (Plan Apo lambda, N.A. 1.4) plus a 1.5× intermediate magnification and a cooled sCMOS camera (Zyla 4.2, Andor Technology).

### 2.6. Statistical Analysis and Interpretation

To identify the effects of the time factor in experiments with a single treatment, one-way repeated measures ANOVA or the equivalent non-parametric test and Dunnett’s multiple comparison post-hoc test were used. In experiments with several treatment groups, two-way repeated measurement ANOVA was used. This was completed by Dunnett’s multiple comparison test to isolate which treatment groups differ from others. In O2050 + Ang II experiments, we supplemented this analysis with a comparison of data obtained at identical time points of the experiments using a t-probe. Maximal Ang II effects in Orlistat + Ang II experiments were compared with a *t*-test. *P* < 0.05 was accepted throughout as a level of significance. Results are expressed as mean ± SEM. Statistical analyses were performed using SigmaStat 3.5 (Systat Software Inc., San Jose, CA, USA).

## 3. Results

### 3.1. CB_1_ Receptors in Rat Cardiac Tissue

**Figure 1:**
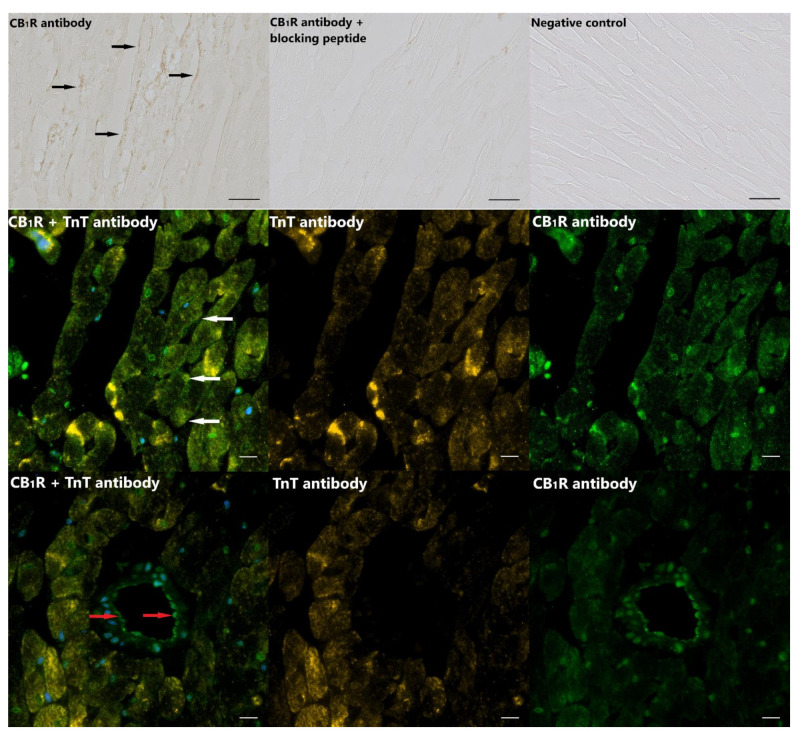
Type-1 cannabinoid receptor (CB_1_R) expression and localization in rat cardiac tissue. Upper panel: Immunohistochemical localization of the CB_1_R protein in cardiac tissue of rats (black arrows). No staining was detected in the absence of the primary antibody (negative control), and a robust decrease in immunostaining was observed when the CB_1_R primary antibody was applied together with blocking peptide; scale bar: 20 µm. Middle and lower panels: Immunofluorescent staining of CB_1_R (green) and cardiac Troponin T (TnT, orange) revealed CB_1_R localization on the sarcolemma of cardiomyocytes (white arrows). CB_1_R staining was also detectable in the wall of vessels (red arrows); scale bar: 10 µm.

Assessment of CB_1_R staining by immunohistochemistry confirmed the expression of CB_1_ receptors in rat cardiac tissue (Figure 1, upper panel). Immunofluorescent visualization of CB_1_R in cardiac tissue sections revealed CB_1_R localization on the sarcolemma of cardiomyocytes (Figure 1, middle and lower panels). Moreover, CB_1_R staining was also detectable in the vascular wall in cross-sectional images of small vessels (Figure 1, lower panel). CB_1_R staining was also present in murine cardiac tissue but was not detectable in hearts of CB_1_R knockout mice (Supplementary Figure S1).

### 3.2. The Effects of Ang II Infusion on Isolated Rat Hearts

**Figure 2:**
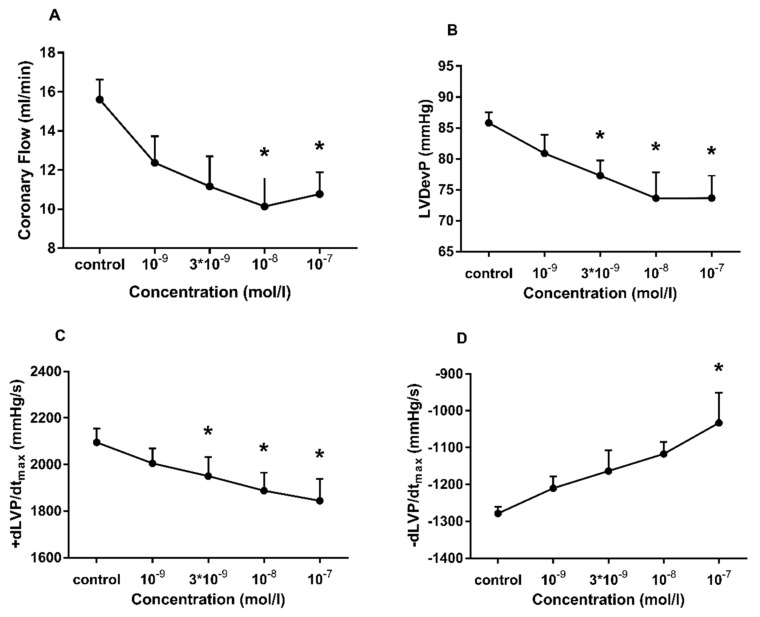
Concentration-dependent effects of angiotensin II (Ang II) on coronary flow (**A**), left ventricular developed pressure (LVDevP) (**B**), +dLVP/dt_max_ (**C**) and −dLVP/dt_max_ (**D**) of isolated rat hearts. In these experiments, Ang II was applied in the range of 10^−9^ to 10^−7^ M. The increasing concentrations of Ang II were infused into the perfusion line consecutively, each for 3 min. Maximal effects measured during the 3-min infusion periods are presented. Mean ± SEM; n = 4; * *p* < 0.05 vs. control (pre-infusion value); one-way repeated measurement ANOVA or ANOVA on ranks and Dunnett’s post-hoc test.

To characterize the effect of Ang II on CF and contractile function, we carried out concentration-response experiments on isolated Langendorff-perfused hearts. Administration of Ang II resulted in a concentration-dependent decrease in CF (Figure 2A). Along with CF deprivation, deterioration of left ventricular contractile performance could be observed, which is evidenced by the declining inotropic (LVDevP, +dP/dt_max_) (Figure 2B,C) and lusitropic (−dP/dt_max_) function (Figure 2D). It is notable that the relative decreases in inotropic and lusitropic function were moderate compared to the relative decline in CF (CF: 31 ± 12%, LVDevP: 14 ± 9%, +dLVP/dt_max_: 12 ± 8%, −dLVP/dt_max_: 19 ± 13% at a concentration of 10^−7^ M).

### 3.3. Effects of Repetitive Ang II Infusion on Coronary Flow and Contractile Function

**Figure 3:**
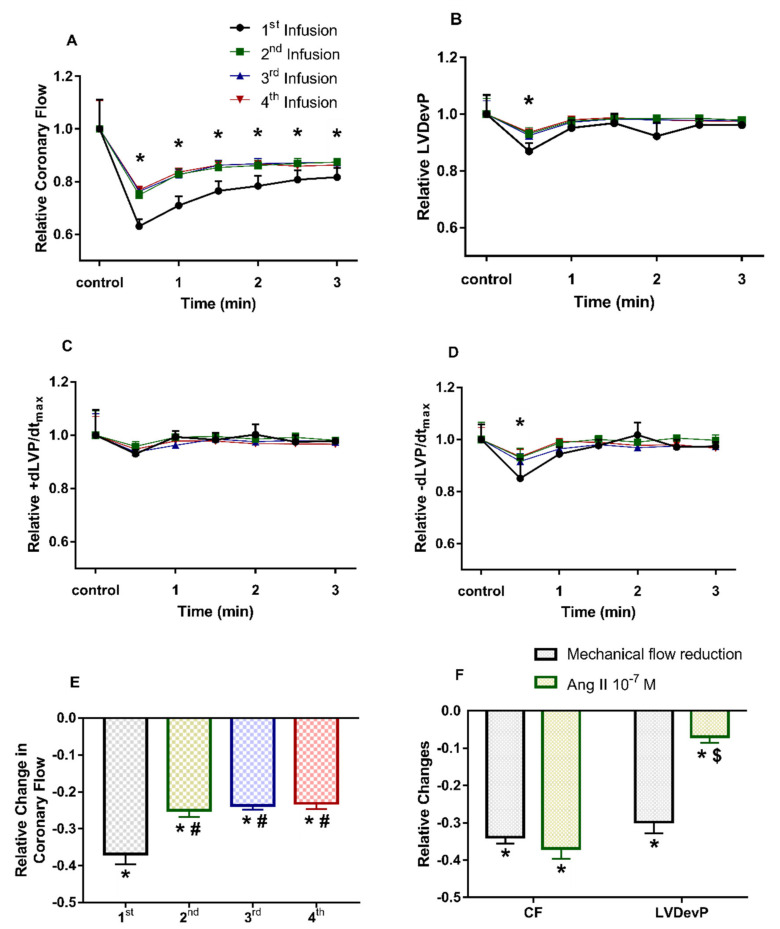
Effects of repetitive angiotensin II (Ang II) infusion on coronary flow (**A**), left ventricular developed pressure (LVDevP) (**B**), +dLVP/dt_max_ (**C**) and −dLVP/dt_max_ (**D**) of isolated rat hearts. Maximal decrease in coronary flow during repetitive infusion periods (**E**). Decrease in LVDevP when the coronary flow is reduced either mechanically by compressing the perfusion line (grey) or by the vasoactive effect of Ang II (green) (**F**). Ang II (10^−7^ M) was administered repetitively to the perfusate of isolated rat hearts (**A**–**E**). Each Ang II infusion period lasted for 3 min and was followed by a 10-min wash-out period. Mean ± SEM; n = 4; * *p* < 0.05 vs. control (pre-infusion value) in each group; one-way repeated measurement ANOVA and Dunnett’s post-hoc test; # *p* < 0.05 vs. first infusion period; one-way ANOVA and Dunnett’s post-hoc test; $ *p* < 0.05 vs. relative decrease in coronary flow, paired *t*-test.

To assess the kinetics of Ang II effects on isolated hearts and to judge the impact of tachyphylaxis on the interpretation of our findings, we infused Ang II (10^−7^ M) repetitively four times. The first administration of Ang II resulted in a more pronounced decline in CF than the subsequent interventions. However, from the second infusion on, the consecutive Ang II effects did not differ from each other significantly (Figure 3A–D). 

As to the kinetics of Ang II effects, it can be observed that at each administration, the decline in CF reached its maximum level in 1 min. This decrease in CF was associated with a moderate decline in LVDevP and lusitropic performance in the first minute of the Ang II administration (Figure 3B,D). However, later during the infusion period LVDevP and −dP/dt_max_ values gradually rose and approached control values in spite of the reduced CF. This might be attributed to the direct inotropic effects of Ang II, because a decrease in CF elicited by compression of the perfusion line entails a decrease of the same magnitude in LVDevP (Figure 3F). 

### 3.4. Effects of CB Receptor Agonists and CB_1_R Antagonist O2050 on Coronary Flow and Contractile Function

**Figure 4:**
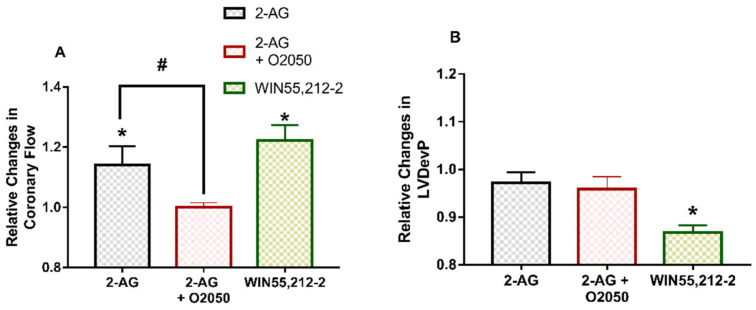
Peak effects of 2-arachydonoyl-glycerol (2-AG) and synthetic CB receptor agonist WIN55,212-2 on coronary flow (**A**) and left ventricular developed pressure (LVDevP) (**B**) of isolated rat hearts. 2-AG (10^−6^ M) and WIN55,212-2 (10^−6^ M) were administered to the perfusate for 5 min. After a 10-min wash-out period, the 2-AG infusion was repeated in the presence of CB_1_R antagonist O2050 (10^−6^ M). Data are presented as relative values compared to pre-infusion control data. Mean ± SEM; n = 9 (2-AG and 2-AG + O2050 experiments) and n = 5 (WIN55,212-2 experiments); * *p* < 0.05 vs. pre-infusion value; # *p* < 0.05 vs. 2-AG peak effect, paired *t*-test.

In separate experiments, we also assessed the effects of 2-AG and synthetic CB receptor agonist WIN55,212-2. Both CB receptor agonists enhanced CF (Figure 4A). 2-AG administration had no significant effect on contractile performance; however, WIN55,212-2 produced a marked decline in LVDevP (Figure 4B). 2-AG was also applied in the presence of CB_1_R neutral antagonist O2050. The CF-increasing effect of 2-AG was prevented by O2050 (Figure 4A). Repeated infusions of 10^−6^ M 2-AG proved to have identical effects in separate experiments (data not shown). We also tested the effects of O2050 infusion alone, and it did not have any significant effect on the cardiac parameters studied (data not shown).

### 3.5. The Influence of CB_1_R Antagonist O2050 and DAGL Inhibitor Orlistat on Ang II Effects

**Figure 5:**
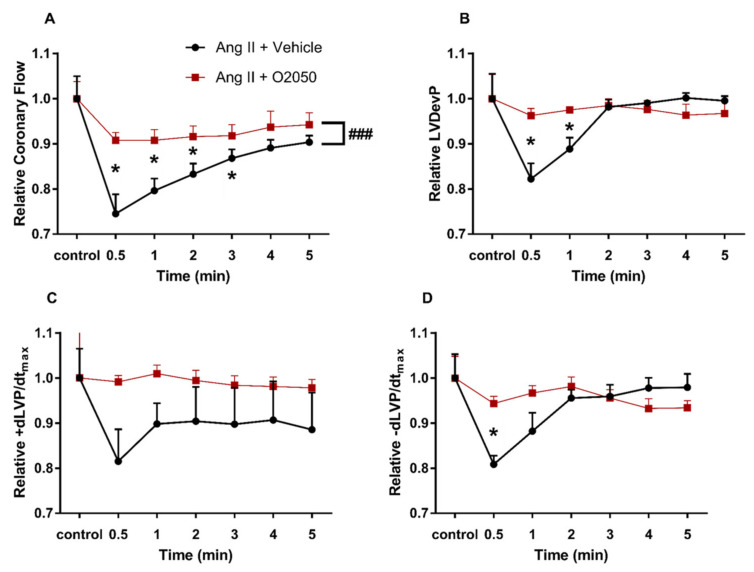
Influence of CB_1_R inhibitor O2050 on the effects of angiotensin II (Ang II) on coronary flow (**A**), left ventricular developed pressure (LVDevP) (**B**), +dLVP/dt_max_ (**C**) and −dLVP/dt_max_ (**D**) of isolated rat hearts. Ang II (10^−7^ M) was administered repetitively to the perfusate. The presented data were recorded during the second infusion period, when Ang II was infused either in the presence of CB_1_R inhibitor O2050 (10^−6^ M) or its vehicle and are expressed as relative values compared to pre-infusion control data. Mean ± SEM; n = 7 & 8; * *p* < 0.05 vs. control (pre-infusion value); ### *p* < 0.001 vs. vehicle; two-way repeated measurement ANOVA and Dunnett’s post-hoc test.

**Figure 6:**
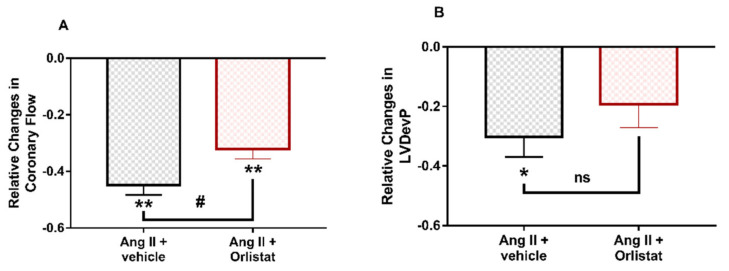
Influence of diacylglycerol-lipase (DAGL) inhibitor Orlistat on the peak effects of angiotensin II (Ang II) on coronary flow (**A**) and left ventricular developed pressure (LVDevP) (**B**) of isolated rat hearts. Ang II (10^−7^ M) was administered repetitively to the perfusate. The presented data were recorded during the second infusion period when Ang II was infused either in the presence of DAGL inhibitor Orlistat (10^−5^ M) or its vehicle and are expressed as relative values compared to pre-infusion control data. Mean ± SEM; n = 4 & 6; * *p* < 0.05 and ** *p* < 0.01 vs. control (pre-infusion value) paired *t*-test; # *p* < 0.05 and ns: non-significant vs. Ang II +vehicle; *t*-test.

To test how 2-AG-release during AT_1_R—G_q/11_ signalling may modify the primary Ang II responses in the heart via potential transactivation of CB_1_ receptors, Ang II was also infused in the presence of CB_1_R antagonist O2050 (Figure 5) and DAGL inhibitor Orlistat (Figure 6). 

The presence of O2050 in the perfusate substantially altered the effects of Ang II. The deep decline in CF was attenuated by O2050 (Figure 5A). In addition, the temporary decrease in inotropic and lusitropic function, which paralleled the decrease in CF during the first 90 s of the ‘Ang II + vehicle’ infusion was completely abolished when CB_1_ receptors were blocked by O2050 (Figure 5B–D). 

Inhibition of DAGL, the enzyme which is supposed to produce 2-AG from DAG during G_q/11_-coupled signalling of Ang II, moderated the Ang II-induced peak reduction in coronary flow (Figure 6A) and the significant decrease in LVDevP was not observable (Figure 6B). 

## 4. Discussion

The main finding of this study is that in an isolated rat heart preparation with intact circulation, the cardiac responses to Ang II are mediated in part by CB_1_ receptor activation, most probably through 2-AG release during angiotensin receptor type-1—G_q/11_ signalling. In our experiments, Ang II induced a concentration-dependent decrease in CF, which was associated with a moderate decrease in cardiac function. These effects were effectively blocked by CB_1_ receptor and DAGL inhibition. Since cardiac CB_1_ receptors were also detected by immunohistochemistry in our experimental setting, this observation proves a functional vascular/hemodynamic effect of Ang II, which is partially exerted via coronary/cardiac CB_1_ receptors. In addition to this observation, direct administration of 2-AG, an endocannabinoid and CB_1_R agonist WIN55,212-2 into the perfusate induced a significant increase in CF, indicating that the endocannabinoid system and CB_1_ receptors play a relevant role in the regulation of cardiac tissue perfusion.

### 4.1. Effects of Endocannabinoids on Cardiac Function 

Endocannabinoids, such as arachidonoyl ethanolamide (anandamide, AEA), 2-AG and 2-arachidonoylglyceryl ether [18,20] are known to have diverse cardiovascular effects, which are attributed mostly to CB_1_ cannabinoid receptors [13,15,23,28,39,40]. Cannabinoids and their synthetic analogues cause vasodilation and hypotension by acting on vascular CB_1_Rs [23,39,41,42]. 

Besides their vascular expression, CB_1_Rs have also been detected in the cardiac tissue by several research groups [43,44] and also by us (Figure 1, and Supplementary Figure S1). Cannabinoid actions in the heart are the result of an interplay between coronary and cardiomyocyte effects. AEA and 2-AG have been shown in various experimental settings to cause coronary vasodilation, increase in coronary blood flow and decrease in cardiac contractility [22,43,45,46,47,48]. In Langendorff hearts, AEA has been reported to cause vasodilation and to decrease LVDevP, effects that are attributed mainly to CB_1_ receptors [49]. Similarly, in vasopressin-pretreated Langendorff hearts, the selective CB_1_ receptor agonist AEA concentration-dependently increased CF [22]. Similar effects were observed with metabolically stable endocannabinoid derivatives, R-methanandamide and noladin ether as well. Moreover, in this study, AEA and 2-AG were detected in the cardiac tissue [22]. In a previous study performed on isolated coronary vessels, we also observed vasodilation of precontracted vessels in response to CB_1_R agonist WIN55,212, an effect that was blocked by CB_1_R antagonists AM251 and O2050 [50]. 

In agreement with literature data, we also found that 2-AG and WIN55,212-2 infusion (Figure 4A) significantly increased CF. The inhibition of 2-AG-induced CF response by O2050 indicates the functional presence of CB_1_R on coronary vessels and suggests that CB_1_ receptors have a significant role in the control of coronary perfusion. Notably, we reported CB_1_Rs on rat coronary vessels in a previous study [50]. The negative inotropic response of the heart to cannabinoids reported by other research groups [40,43,49] was observed only during WIN55,212-2 administration in our study, and did not develop when 2-AG was applied (Figure 4B). This discrepancy can be explained by the fast metabolism of 2-AG, which may not allow a build-up of 2-AG concentration in the close environment of cardiomyocytes which is high enough to elicit the negative inotropy when intracoronary application is used. On the other hand, we need to take into account that the observed 2-AG-induced rise in CF evokes a consequential positive effect on contractility, which can mask its direct negative inotropic action. 

### 4.2. Role of Angiotensin II-Induced Endocannabinoid Release in Cardiac Function

We have previously reported that Ang II via AT_1_ receptors induces the release of 2-AG in the vascular tissue, which decreases Ang II-induced vasoconstriction [13,15]. Signalling-induced endocannabinoid release via activation of G protein-coupled receptors (GPCRs) and downstream phospholipase C-activation-mediated mechanisms have been detected first in the neural tissue serving as retrograde synaptic inhibition [20]. Endocannabinoid release has also been detected by bioluminescence resonance energy transfer method in isolated cell systems in response to calcium-signal-generating GPCR agonists, such as Ang II via AT_1_ receptors, and acetylcholine via muscarinic receptors [12,51]. GPCR agonist-induced release of 2-AG has also been reported in the vascular tissue previously [47,52]. Since CB_1_ receptors have also been detected in the cardiac tissue, we can postulate that Ang II-induced 2-AG release may also have relevance in the heart. 

In our study, Ang II concentration-dependently decreased coronary perfusion flow with a moderate negative inotropic and lusitropic effect in Langendorff rat hearts (Figure 2). This is in agreement with the findings of van Esch et al. [4], but seems to contradict other studies which reported positive inotropy at higher Ang II-concentrations [5] and in isolated cardiomyocytes [7,8]. However, in our experimental setting, the negative functional effects on the heart are suggested to be an indirect consequence of decreased coronary perfusion. In our system, interventions, which decrease CF without having a direct influence on cardiac cells (e.g., mechanical compression of the inflow line (Figure 3F), or vasoconstrictors with mere vascular effects [33]), normally elicit a decrease of the same magnitude in contractile function. In the current study, CF decreased and remained lower than control throughout the Ang II administration (Figure 3A); however, this was accompanied by only a moderate and transient decrease in contractile performance (Figure 3B–D). This may suggest that a positive inotropic effect was present but could only mitigate the detrimental functional effects of the simultaneous strong coronary vasoconstriction. 

The multiple administrations of Ang II into the coronary perfusion resulted in desensitization, in which the effect of the 2nd administration was diminished compared to the first one but did not differ from the further ones (Figure 3). Therefore, we applied Ang II pretreatment to prevent tachyphylaxis and used the 2nd Ang II infusion to assess the presence of CB_1_ receptor transactivation. We suggest that this setting is the most compliant to the in vivo situation. Tachyphylaxis induced by Ang II has also been excluded similarly by others [53]. 

In our experiments, we found that CB_1_ receptor blockade by O2050 (Figure 5) abolished, and inhibition of DAGL attenuated (Figure 6) Ang II-induced CF reduction and cardiac effects. This seems to contradict previous studies on isolated coronary arteries and also on the aorta, which reported augmented vasoconstriction in response to Ang II when CB_1_R inhibitor O2050 was applied [15,50]. However, it is important to note that the Langendorff-perfused heart is a more complex system in which the applied pharmacons act on the cardiac tissue and on the coronary vessels as well, influencing CF and cardiac function in a complex manner. In the intact heart, CF is primarily controlled by the oxygen demand of cardiac work. This regulatory phenomenon often overwrites the direct vascular effects of vasoactive agents and may explain why we observed a CF effect that is opposite to the one expected on the basis of isolated vessel studies. This phenomenon can be most probably explained by the altered cardiac effects of Ang II upon CB_1_R inhibition. Specifically, we propose that O2050 blocks the CB_1_R-induced negative inotropic effect, which allows the Ang II-induced positive inotropy to develop (which is otherwise masked by flow deprivation). This increases the overall oxygen demand and thus the CF, and overweighs the CF-reducing effect of the loss of direct vasodilator 2-AG action.

Although we did not observe negative inotropy upon 2-AG administration to the coronary perfusate, it was evident when WIN55,212-2 was applied (Figure 4A). The lack of 2-AG effect may be caused by its fast metabolism, which may prevent the build-up of a concentration in the cardiac tissue upon intracoronary application, which is sufficiently high to exert this effect. However, we may assume that when 2-AG is released from cardiomyocytes during Ang II signalling, negative inotropy develops as a paracrine/autocrine effect. 

**Figure 7:**
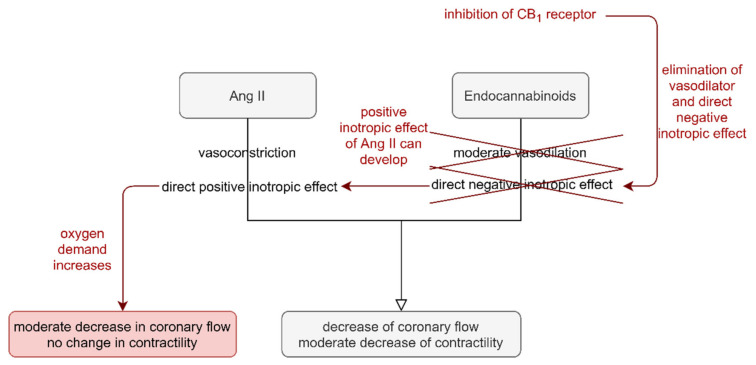
Postulated mechanism of the role of angiotensin II-induced (Ang II) 2-arachidonoylgycerol (2-AG) release in Ang II-induced short-term cardiac effects. Ang II causes vasoconstriction of the coronaries and has positive inotropic effects on cardiomyocytes. On the other hand, 2-AG release during Ang II signalling causes moderate vasodilation and has negative inotropic effects. The summation of these opposing effects manifests in diminished coronary flow (CF) accompanied by a moderate decrease in contractility in the Langendorff-perfused rat heart (black arrows). Inhibition of CB_1_ receptor, and 2-AG generation eliminates the counteraction of 2-AG on the positive inotropic effect of Ang II. The unopposed positive inotropic effect of Ang II enhances cardiac oxygen demand, which counterregulates the vasoconstrictor effect of Ang II. As a result, the flow-reducing effect of Ang II is moderated, and the opposing effects of diminished CF and increased contractility cancel each other out, leaving the contractile function unaltered (red arrows).

In summary, the diminished CF-reducing effect of Ang II during DAGL blockade and CB_1_R inhibition can be attributed to the enhanced cardiac oxygen demand, which is evoked by increased cardiac contractility. This overrides Ang II-induced vasoconstriction. Thus, our observation suggests that the acute effects of Ang II on isolated Langendorff hearts are modulated by PLC-signalling-induced endocannabinoid release via CB_1_R stimulation. We propose that the short-term cardiac effects of Ang II are exhibited as a delicate balance of the simultaneous cardiac and vascular effects of Ang II and 2-AG (Figure 7).

## 5. Conclusions

In this study, we report that the short-term effects of Ang II in an isolated heart preparation with intact circulation are modulated by DAGL activation and CB_1_ receptor transactivation, indicating a significant role of Ang II-signalling-induced release of the endocannabinoid 2-AG. We propose that the short-term cardiac effects of Ang II are exhibited as the summation of the simultaneous cardiac and vascular effects of Ang II and 2-AG.

Our study has proved that short-term Ang II effects are partially mediated by CB_1_ receptor activation. Whether this holds for long-term effects of Ang II, when the local renin-angiotensin system is upregulated in the heart under pathological conditions, and whether these potential effects are detrimental or beneficial needs further investigation. 

## Data Availability

The data presented in this study are openly available in Mendeley Data at https://data.mendeley.com/drafts/7vy77nx4yj, accessed on 25 January 2021.

## Supplementary Materials

The following are available online at https://www.mdpi.com/2073-4409/10/4/724/s1, Figure S1: CB_1_ receptor (CB_1_R) immunostaining in wild type and CB_1_R knockout murine cardiac tissue. Figure S2: Concentration-dependent effects of 2-arachidonoylglycerol (2AG) on coronary flow and left developed ventricular pressure (LVDevP) of isolated rat hearts.
