# Supplementary material for: Angiotensin II-Induced Cardiac Effects Are Modulated by Endocannabinoid-Mediated CB1 Receptor Activation
Source: Cells. 2021 Mar 24;10(4):724. doi: 10.3390/cells10040724 (PMC8064086; doi:10.3390/cells10040724)
Supplement: Supplementary file 1 [file cells-10-00724-s001.pdf]

Supplementary

1. *CB_1 receptor expression in mouse cardiac tissue*

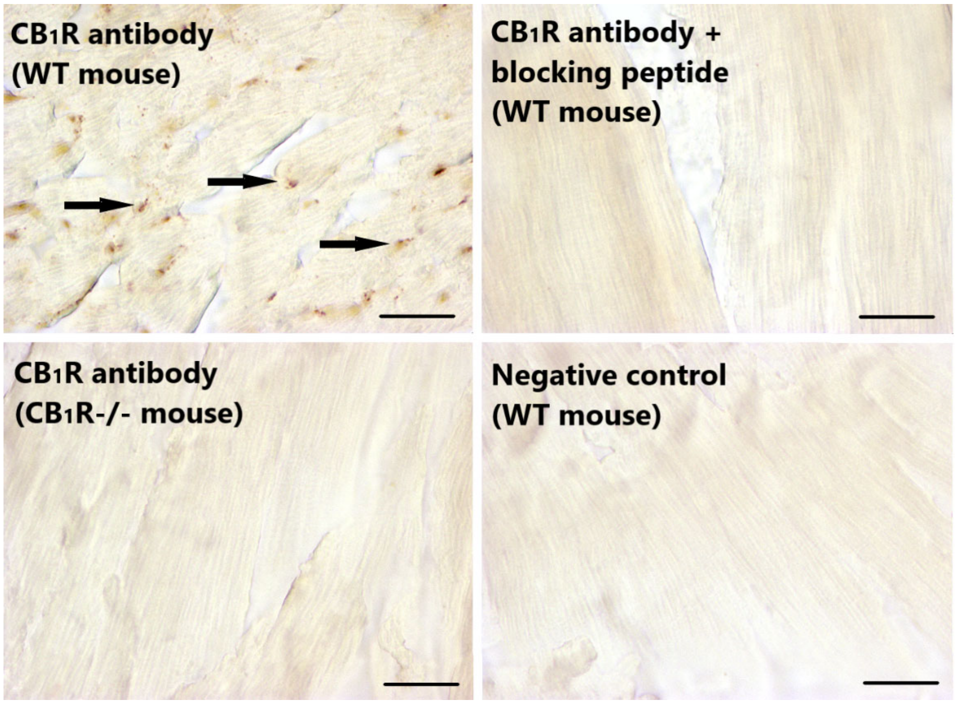

Suppl. Figure S1. CB_1 receptor (CB_1R) immunostaining in wild type and CB_1R knockout murine cardiac tissue. In wild type (WT) hearts CB_1R immunostaining confirmed CB_1R expression in mouse cardiac tissue (black arrows). No staining was detected in the absence of the primary antibody (negative control) and a robust decrease in immunostaining was observed when the CB_1 primary antibody was applied together with blocking peptide. CB_1R staining was undetectable in hearts of CB_1R knockout (CB_1-/-) mice, suggesting high specificity of the applied CB_1R antibody (CB1 Receptor Polyclonal Antibody (rabbit), Cayman Chemicals, Ann Arbor, USA) Scale bar: 100 μm.

2. *Effects of CB agonist 2-AG*

To assess the effects of CB agonist 2-AG on cardiac function, it was infused to the perfusate of isolated hearts in incresing concentrations (10^{-7}, $3x10^{-7}$, 10^{-6} M), each concentration was administered for 3 min. A moderate rise in coronary flow and a slight decrease in contractile function (developed pressure, LVDevP) could be observed at the highest concentration applied. However, these changes did not reach the level of significance (Suppl. Figure 2).

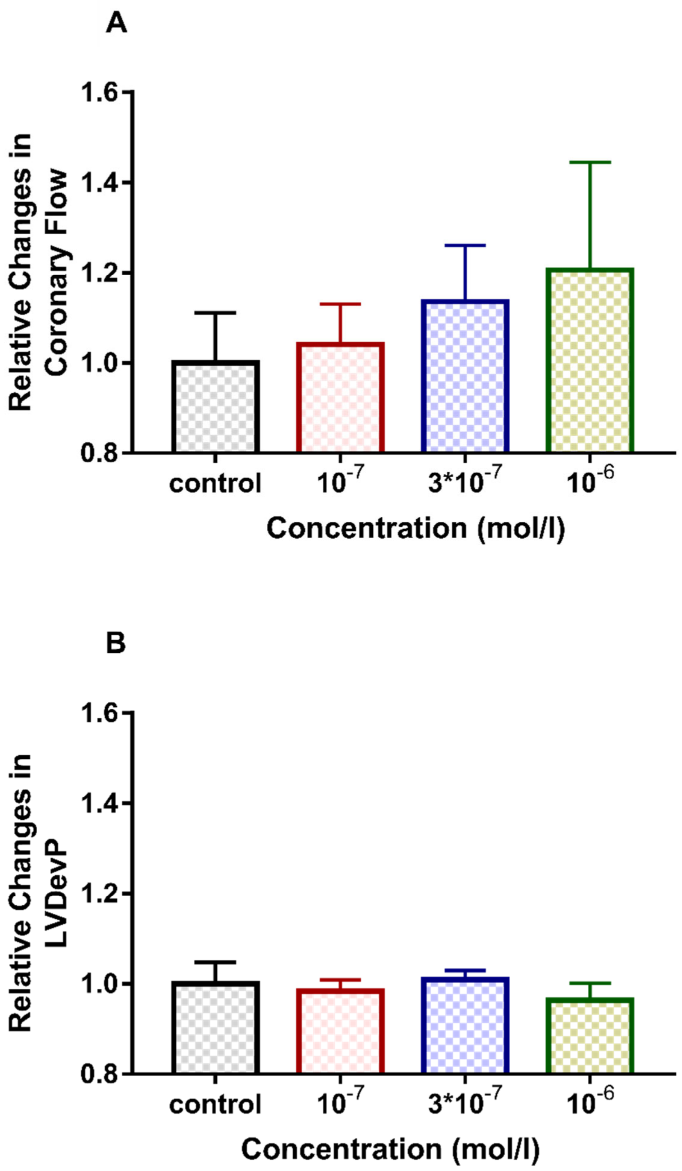

Suppl. Figure S2. Concentration-dependent effects of 2-arachidonoylglycerol (2-AG) on coronary flow **(A)** and left ventricular developed pressure (LVDevP) **(B)** of isolated rat hearts. In these experiments 2-AG was applied in a range of 10^{-7} to 10^{-6} M. The increasing concentrations of 2-AG were infused into the perfusion line consecutively, each for 3 min. Maximal effects during the 3-min infusions are presented. Mean ± SEM; *n*=4; p values are greater than 0.05 vs. control (pre-infusion value); one-way repeated measurement ANOVA
